# Alkylated Sesamol Derivatives as Potent Antioxidants

**DOI:** 10.3390/molecules25143300

**Published:** 2020-07-21

**Authors:** Ivanete C. Palheta, Lanalice R. Ferreira, Joyce K. L. Vale, Osmarina P. P. Silva, Anderson M. Herculano, Karen R. H. M. Oliveira, Antonio M. J. Chaves Neto, Joaquín M. Campos, Cleydson B. R. Santos, Rosivaldo S. Borges

**Affiliations:** 1 Núcleo de Estudos e Seleção de Moléculas Bioativas, Instituto de Ciências da Saúde, Universidade Federal do Pará, Belém 66075-110, Brazil; lanalicerodrigues30@gmail.com (L.R.F.); joy.farmc@gmail.com (J.K.L.V.); opps@ufpa.br (O.P.P.S.); 2 Programa de Pós-Graduação em Química Medicinal e Modelagem Molecular, Instituto de Ciências da Saúde, Universidade Federal do Pará, Belém 66075-110, Brazil; aherculanos@gmail.com (A.M.H.); oliveirakrm@gmail.com (K.R.H.M.O.); breno@unifap.br (C.B.R.S.); 3 Faculdade de Física, Instituto de Ciências Exatas e Naturais, Universidade Federal do Pará, Belém 66075-110, Brazil; antmchaves@yahoo.com.br; 4 Department of Pharmaceutical Organic Chemistry, University of Granada, 18071 Granada, Spain; jmcampos@ugr.es; 5 Laboratorio de Modelagem e Química Computacional—LMQC, Federal University of Amapá. Rod. Juscelino Kubitschek, Km 02, Macapá 68902-280, Brazil

**Keywords:** antioxidant capacity, sesamol, DFT, electron transfer, hydrogen transfer

## Abstract

Sesamol is a phenolic derivative. Its antioxidant activity is low than that of Trolox and depends on benzodioxole moiety. Thus, a molecular modification strategy through alkylation, inspired by natural and synthetic antioxidants, was studied by molecular modeling at the DFT/B3LYP level of theory by comparing the 6-31+G(d,p) and 6-311++G(2d,2p) basis sets. All proposed derivatives were compared to classical related antioxidants such as Trolox, *t*-butylated hydroxytoluene (BHT) and *t*-butylated hydroxyanisole (BHA). According to our results, molecular orbitals, single electron or hydrogen-atom transfers, spin density distributions, and alkyl substitutions at the ortho positions related to phenol moiety were found to be more effective than any other positions. The trimethylated derivative was more potent than Trolox. *t*-Butylated derivatives were stronger than all other alkylated derivatives and may be new alternative forms of modified antioxidants from natural products with applications in the chemical, pharmaceutical, and food industries.

## 1. Introduction

Free radical species are molecules that contain one or more unpaired electrons with short half-life, low stability, and high chemical reactivity [1]. They are produced naturally or through some biological dysfunction, and they are involved in obtaining energy, phagocytosis, the regulation of cell growth, the synthesis of substances, intercellular signaling, oxidative stress, and lipid peroxidation [2].

Oxidative stress is considered a vulnerability status of a defense system involving the overproduction of free radicals overcoming endogenous antioxidant capacities. The excess of these compounds in the body, especially superoxide anions (O_2_^−•^) and peroxyls (RO_2_^•^), causes damage to proteins, lipids, and DNA, as they are involved in several processes including aging, atherosclerosis, cancer, heart diseases, and lung problems [3]. These pathophysiological processes can be reversed when an antioxidant such as ascorbic acid, α-tocopherol, resveratrol, catechins, and glutathione overcome oxidative damage [4]. Several studies have aimed to increase antioxidant activity, solubility, bioavailability, and selective toxicity via molecular modifications in natural compounds [3]; however, few efforts have been focused on sesamol.

**Figure 1:**
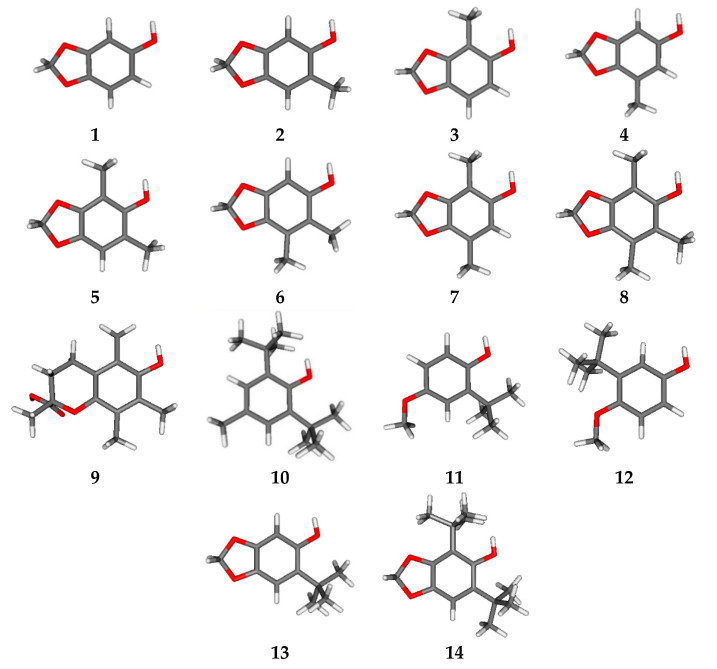
Optimized structure of sesamol, related alkylated derivatives, and classical antioxidants by the B3LYP/6-31+G(d,p) basis set (carbon = gray; hydrogen = white; and oxygen = red).

Sesamol **1** is a phenolic antioxidant that inhibits radical reactions, inactivating reactive oxygen species. It also had anticancer, antimutagenic, and hepatoprotective activities [5,6,7]. Antioxidant activity basically involves several mechanisms; among them, the most important are the single electron transfer (SET) and the hydrogen atom transfer (HAT) to a free radical, generating a derivative or intermediate radical neutralized by the resonance of an aromatic ring [8,9]. These mechanisms have already been connected to the antioxidant action of sesamol and its derivatives through the diphenyl picrylhydrazyl radical (DPPH) [10] with a reduced theoretical capacity when compared to tocopherol derivatives [11]. Therefore, this work aimed to increase the antioxidant capacity of sesamol by alkylation (**2**–**8**, **13**, and **14**) inspired by tocopherol derivatives and classic antioxidants such as trolox **9**, *t*-butylated hydroxytoluene **10** (BHT), and *t*-butylated hydroxyanisole **11** and **12** (BHA) (Figure 1).

## 2. Results and Discussion

### 2.1. Structure-Antioxidant Capacity by Electron or Hydrogen Transfers

The optimized chemical structures of sesamol, their related alkyl-derivatives, and classical antioxidants by the B3LYP/6-31+G(d,p) basis set are shown in Figure 1. The results of *E*_HOMO_, *E*_LUMO_, gap energy, ionization potential (IP), SET, bond dissociation energy of the phenol moiety (BDE_OH_), and HAT in the B3LYP/6-31+G(d,p) level for sesamol **1**, alkylated derivatives (**2**–**8**, **13**, and **14**), Trolox **9**, BHT **10**, and BHA (**11** and **12**) are exposed in Table 1. The proposed compounds by the addition of methyl or *t*-butyl groups were compared to each other using electron and hydrogen-atom transfer calculations.

Stabilization energy is a simplified method for predicting the capacity to trap free radicals from phenolic derivatives by theoretical calculations using SETs or HATs. The relative stability for radicals at specified positions and major portions was related by the sesamol values, as shown in Table 1.

All negative values are related to better antioxidant compounds when compared to sesamol. Alkylation increases these values in any position on a sesamol structure. Nonetheless, all derivatives substituted at the ortho positions are more potent than substituted derivatives at the meta positions. Double or triple alkylation improves SET and HAT values due to resonance effects between the aromatic system and phenoxyl for the stabilization of cation-free radicals or semiquinone forms.

The electron transfer capacity is also expressed by the IP of the compounds, as shown in Table 1. All studied derivatives had a lower IP than sesamol (174.31 kcal/mol), and, therefore, they had a better antioxidant potential. Derivative **8** (163.11 kcal/mol) exhibited the best value of the IP, exceeding all alkylated derivatives, including the classic antioxidants Trolox **9** (163.35 kcal/mol), BHT **10** (173.15 kcal/mol), and BHA **11** and **12** (171.40 and 171.52 kcal/mol) for electron transfers, showing a similar ability for free radical interaction and elimination by electron transfer mechanism.

Likewise, low values for the BDE_OH_ are related to the higher antioxidant capacity after a hydrogen atom transfer process, suggesting a better antioxidant capacity [12]. An additional methyl or *t*-butyl group decreased BDE_OH_ when compared to sesamol (83.03 kcal/mol). The derivatives **4**, **8**, and **14** (79.07, 78.59, and 75.88 kcal/mol, respectively), showed lower BDE_OH_ values than Trolox **9** (79.24 kcal/mol), having the best HAT values among all proposed sesamol derivatives.

According to SET, the trimethoxylated **8** (−11.20 kcal/mol) and di-*t*-butylated derivatives **14** (−9.20 kcal/mol) showed smaller values and better antioxidant capacities compared to sesamol. However, the higher HAT values for the di-*t*-butylated derivative **14** (−7.15 kcal/mol) are important signifiers for hydrogen scavenger capacity. These results showed that di-*t*-butylization is the most important strategy for the design and development of novel sesamol derivatives with antioxidant potential.

### 2.2. Structure-Chemical Reactivity of Alkylated Sesamol Derivatives

The energy difference (GAP) between LUMO and HOMO orbitals determines the chemical reactivity of a molecule. Trolox **9** was more nucleophilic than sesamol **1**, with their HOMO values equaling −5.38 and eV −5.58 and GAP values corresponding to 5.89 and 5.09 eV, respectively. The trimethyl derivative **8** (−5.25 eV) showed a higher value than Trolox **9**, BHT **10**, and BHA (**11** and **12**), for nucleophilicity, exhibiting superior electron transfer abilities. Alkylation modifications did not significantly reduce the GAP value between HOMO and LUMO. In fact, no compounds were superior than Trolox **9** (5.89 eV), and most alkylated analogs were superior to sesamol **1** (5.09 eV).

In addition, in antioxidant compounds, the high electron density distribution of HOMO orbitals determines the most likely sites that can be easily attacked by free radicals [13]. The HOMO arrangement showed a greater influence on the ether and phenol moieties, which were the main functional groups responsible for the increase of the resonance structures. However, alkyl moieties had additional influences and all alkylated compounds had similar performances.

**Figure 2:**
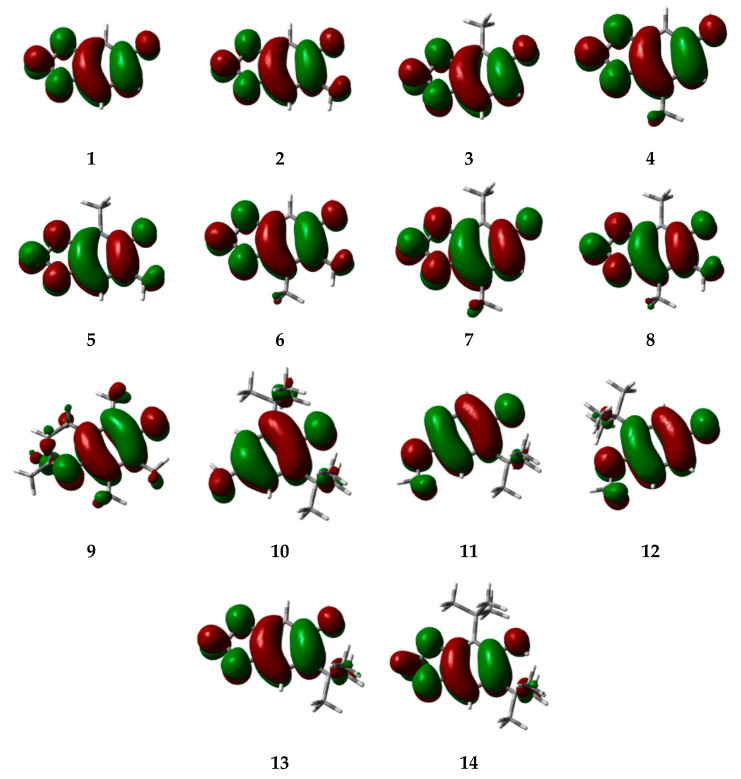
HOMO structure for sesamol, related alkylated derivatives, and classical antioxidants by B3LYP/6-31+G(d,p). 3D models of HOMO coefficients are in green and red.

The influence of alkyl groups on the nucleophilicity of these compounds was much smaller on the meta positions but significant on the para positions of the ring. However, the *ortho* orientations had strong participation in the π conjugation between both substituents. This behavior could be increased by the participation of the ether groups at the *meta*- and *para*- positions. These resonance effects can be observed in Figure 2.

Previously, the influence of alkyl groups in *ortho* positions was thought to be an important factor for high scavenging activity [13]. The ether group orientations have affected the extension of the π system by changing its energy according to the hydroxy and alkylated positions. As a consequence, molecules that showed several resonance structures were more stable and had higher SET and HAT values. The expressive influence of alkyl and ether moieties did not allow for electronic conjugation between the heterocyclic ring and alkyl groups. Nonetheless, all molecules were characterized by a limited conformation flexibility and the fact that SET and HAT values especially depend on heterocycle rings. Therefore, our proposed derivatives, especially those that were di-*t*-butylated, may be alternative modified antioxidants of natural products with applications in the chemical, pharmaceutical, and food industries.

### 2.3. Structure-Chemical Stability of Alkylated Sesamol Derivatives

The chemical stability of free radicals can be characterized by spin density distributions. The energy of a radical decreases when unpaired electron π delocalization occurs after an electron transfer and especially after a hydrogen abstraction as we can see in semiquinone forms. In fact, semiquinones are more stable than cation-free radicals [14].

**Figure 3:**
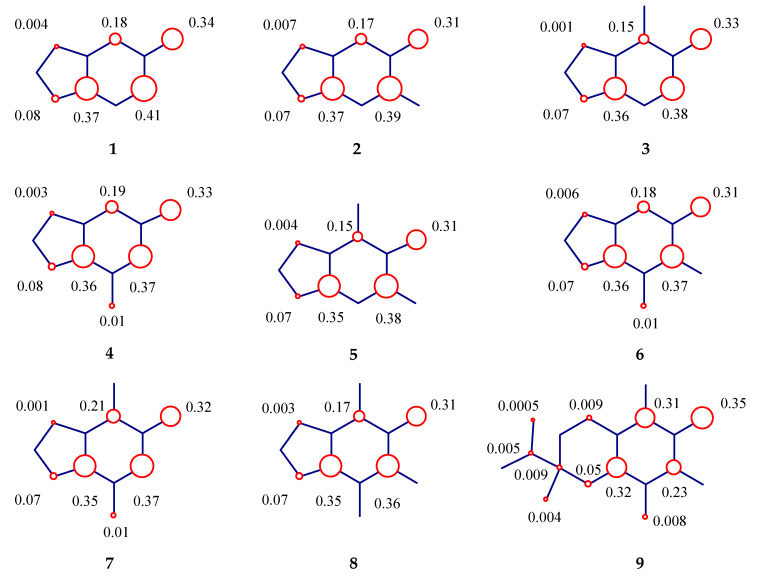

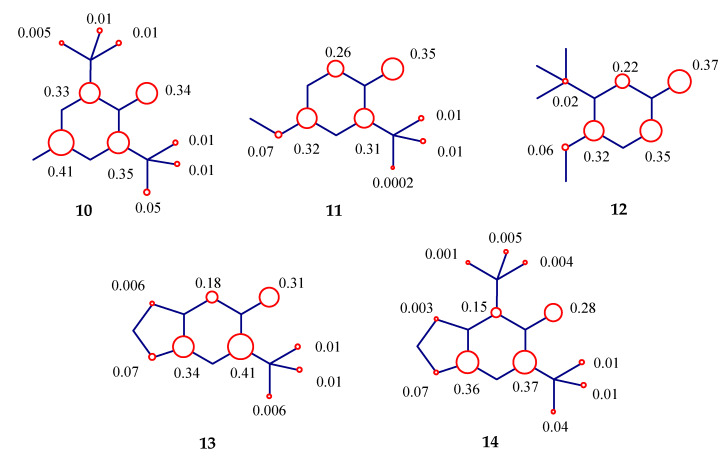
Spin density contributions of sesamol, related alkylated derivatives, and classical antioxidants by B3LYP/6-31+G(d,p). The explored spin density values were calculated by considering the hydrogen uptake from the phenolic hydroxyl group and carbons from the benzene portion.

In Figure 3, the calculated spin density contributions in regards to hydrogen abstraction of the phenolic hydroxyl group shows that the influence of phenolic oxygen contribution was proportional to antioxidant and chemical stability. The same behavior was observed on carbons of the benzene moiety. The phenoxyl group compounds, the spin density contributions were between 0.37 and 0.28 and more stable than sesamol **1** (0.34), Trolox **9** (0.35), BHT **10** (0.34), and BHA **11** and **12** (0.35 and 0.37, respectively). The presence of phenolic oxygen in our alkylated derivatives had a small influence (a range from 0.33 to 0.28), especially for the main compound, the di-*t*-butylated derivative **14** (0.28). Thus, the lowest spin density contributions in oxygen of phenoxyl groups was related to the most stable semiquinone forms, and the number of contributions on benzene rings was the second lowest.

### 2.4. Comparative Study between B3LYP/6-31+G(d,p) and B3LYP/6-311++G(2d,2p)

Table 2 shows the results for derivatives of *E*_HOMO_, *E*_LUMO_, gap energy, SET, and IP using the B3LYP/6-311++G(2d,2p) level of theory. All calculated IP values in B3LYP/6-31+G(d,p) were compared to B3LYP/6-311++G(2d,2p).

By comparing the values, we can observe that the highest standard deviation values were found for compounds **6** and **7**. However, when we compared the IP values between these two base sets, we could see that there was no change in the order among our three main compounds with high antioxidant potencies—**8**, **9**, and **14**. Thus, the double zeta could be applied in this case and be a useful, simple, fast, and easy methodology for antioxidant selection when compared to data from the literature [15].

## 3. Materials and Methods

All proposed derivatives are shown in Figure 1, where each molecular modification on sesamol followed the same structural patterns with one, two, and three methylations similar to synthetic α-tocopherol derivatives (Trolox). In addition, BHA and BHT have one and two di-*t*-butylated, respectively. The energy minimization for all structures was obtained through B3LYP/6-31+G(d,p) calculations. The relationship between chemical structure and antioxidant capacity relationship was compared using the double zeta and triple zeta B3LYP/6-311++G(2d,2p) at the computational level [16,17,18,19]. Excellent results were previously reported for sesamol-related derivatives [11,20,21]. All molecular calculations, including the visualization of the spin density distribution, were achieved using Gaussian 09 [22]. Chemical reactivity was evaluated by HOMO and LUMO energies, which were related to nucleophilicity and electrophilicity parameters, respectively [23]. The chemical reactivity was obtained by the Gap^LUMO−HOMO^ difference (see Equation (1) and the spin densities.

Gap = *E*_LUMO_ − *E*_HOMO_ (1)

The IP and the BDE_OH_ were related to electron or hydrogen donations, respectively. The IP was calculated as the energy difference between the neutral molecule (ArOH) and the respective cation-free radical (ArOH^•+^), as shown in Equation (2). The BDE_OH_ was calculated as the energy difference between the neutral molecule (ArOH) and its respective semiquinone (ArO^•^) plus a hydrogen radical (H^•^), as shown in Equation (3). The radical stability for electron or hydrogen transfers were determined by related stabilization energy, as shown in Equation (4), for single electron transfers (SETs) and for hydrogen atom transfers (HATs), as shown in Equation (5). All SET and HAT mechanisms are related to sesamol (SOH). For all other calculations, including the IP and BDE of sesamol derivatives, ArOH was used for all phenol derivatives studied in this work.

IP = *E*_ArOH_^•+^ − *E*_ArOH_ (2)

BDE_OH_ = (*E*_ArO_^•^ + *E*_H_^•^) − *E*_ArOH_ (3)

SET = [*E*_ArOH_^•+^ + *E*_SOH_] − [*E*_ArOH_ + *E*_SOH_^•+^] (4)

HAT = [*E*_ArO_^•^ + *E*_SOH_] − [*E*_ArOH_ + *E*_SO_^•^] (5)

## 4. Conclusions

Alkylated modifications were found to generally increase the free radical scavenging activity of sesamol. Our results showed that prevalent involvement of alkylated groups on HOMO, IP, BDE_OH_, SET, HAT, and spin densities. The trimethylated (**8**) and *t*-butylated (**14**) analogs of sesamol showed the best theoretical results, exceeding the values for the classic antioxidants Trolox, BHT, and BHA. The introduction of alkyl groups led to the largest free radical stability and an increase of resonance structures when compared to sesamol. The presence of strong π-type electronic system found in the spin densities may explain the potent antioxidant activity of the alkylated sesamol derivatives. Our proposed derivatives, especially the *t*-butylated derivative (**14**), may be new alternatives for modified antioxidants from natural products with applications in the chemical, pharmaceutical, and food industries.

## Figures and Tables

**Table 1:** Theoretical properties of sesamol and related derivatives using B3LYP/6-31+G(d,p). HOMO: highest occupied molecular orbital; LUMO: lowest unoccupied molecular orbital; GAP: energy difference; IP: ionization potential; SET: single electron; BDE_OH_: bond dissociation energy of the phenol moiety; and HAT: hydrogen atom transfer.

Derivatives	HOMO (eV)	LUMO (eV)	GAP (eV)	IP (kcal/mol)	SET (kcal/mol)	BDE_OH_ (kcal/mol)	HAT (kcal/mol)
**1**	−5.58	−0.49	5.09	174.31	0.00	83.03	0.00
**2**	−5.42	−0.39	5.03	168.98	−5.33	80.77	−2.26
**3**	−5.49	−0.27	5.22	171.16	−3.15	81.24	−1.79
**4**	−5.48	−0.30	5.18	170.79	−3.52	82.64	−0.39
**5**	−5.34	−0.25	5.08	166.06	−8.25	79.07	−3.39
**6**	−5.33	−0.20	5.13	165.91	−8.40	80.35	−2.68
**7**	−5.39	−0.22	5.17	167.79	−6.52	80.79	−2.24
**8**	−5.25	−0.23	5.02	163.11	−11.20	78.59	−4.44
**9**	−5.38	−0.48	5.89	163.35	−10.96	79.24	−3.79
**10**	−5.78	−0.19	5.60	173.15	−1.16	80.53	−2.50
**11**	−5.60	−0.38	5.22	171.40	−2.91	81.93	−1.10
**12**	−5.60	−0.38	5.22	171.52	−2.79	84.19	1.16
**13**	−5.46	−0.38	5.07	167.70	−6.61	80.17	−2.86
**14**	−5.44	−0.25	5.19	165.11	−9.20	75.88	−7.15

**Table 2:** Theoretical properties of sesamol and related derivatives using B3LYP/6-311++G(2d,2p).

Derivatives	HOMO (eV)	LUMO (eV)	GAP (eV)	SET (kcal/mol)	IP (kcal/mol)	IP * (kcal/mol)	Average (kcal/mol)	SD **
**1**	−5.59	−0.51	5.07	0.00	174.46	174.31	174.38	0.106
**2**	−5.43	−0.42	5.00	−5.42	169.04	168.98	169.01	0.042
**3**	−5.49	−0.44	5.05	−3.22	171.24	171.16	171.2	0.056
**4**	−5.49	−0.33	5.16	−3.60	170.86	170.79	170.82	0.049
**5**	−5.34	−0.45	4.89	−8.40	166.06	166.06	166.06	0.000
**6**	−5.44	−0.37	5.07	−6.11	168.35	165.91	167.13	1.725
**7**	−5.48	−0.43	5.04	−4.82	169.63	167.79	168.71	1.301
**8**	−5.25	−0.44	4.81	−11.44	163.02	163.11	163.06	0.063
**9**	−5.40	−0.50	4.89	−10.82	163.64	163.35	163.49	0.205
**10**	−5.81	−0.37	5.43	−0.98	173.48	173.15	173.31	0.233
**11**	−5.63	−0.42	5.20	−2.65	171.81	171.40	171.60	0.289
**12**	−5.63	−0.42	5.21	−2.46	172.00	171.52	171.76	0.339
**13**	−5.46	−0.41	5.05	−6.81	167.65	167.70	167.67	0.035
**14**	−5.43	−0.39	5.03	−9.91	164.55	165.11	164.83	0.395

* IP value in B3LYP/6-31+G(d,p). ** SD = Standard Deviation.
